# Microsatellite marker development based on next-generation sequencing for the smooth marron (*Cherax cainii*, Austin) and cross-species amplification in other *Cherax* species

**DOI:** 10.1186/s13104-015-1345-z

**Published:** 2015-08-25

**Authors:** Shannon R. Loughnan, Luciano B. Beheregaray, Nicholas A. Robinson

**Affiliations:** Molecular Ecology Laboratory, School of Biological Sciences, Flinders University, PO Box 2100, Adelaide, 5001 SA Australia; Nofima, PO Box 5010, N-1432 Ås, Norway

**Keywords:** Marron, *Cherax cainii*, *Cherax tenuimanus*, Microsatellites, Next-generation sequencing

## Abstract

**Background:**

The smooth marron, *Cherax cainii* is an important freshwater crustacean species for aquaculture and for a local wild fishery. *C. tenuimanus*, commonly known as the hairy marron is under threat from environmental impacts and genetic introgression from *C. cainii* that is hampering the survival of wild *C. tenuimanus* stocks. Marron are endemic to the south-west of Western Australia and *C. tenuimanus* is restricted to only the Margaret River.

**Results:**

To isolate microsatellite sequences, shotgun 454 pyrosequencing was performed resulting in 184,981 DNA sequence reads. Following screening for microsatellites, 8799 putative microsatellite loci were detected and PCR primers were designed for 968 of these. Ten microsatellite loci were screened in 30 captive *C. cainii* individuals with eight loci producing unambiguous results. The average number of alleles per locus was 4.7 and average *H*_*e*_ was 0.474. Following an analysis of relatedness, 79 % of captive dyads were assigned as unrelated. Utilising *C.quadricarinatus* and *C. destructor*, cross-species amplification tests were conducted and amplification was achieved at four of the eight loci.

**Conclusions:**

Using next-generation sequencing methods, eight polymorphic microsatellite loci were developed from *C. cainii*, with potential for cross amplification in other *Cherax* species. The markers can be utilised for studies of natural genetic stock structure and for monitoring relatedness levels and genetic variation in both wild and captive populations.

**Electronic supplementary material:**

The online version of this article (doi:10.1186/s13104-015-1345-z) contains supplementary material, which is available to authorized users.

## Findings

Commonly known as the smooth marron, *Cherax cainii* (Austin) is a riverine crustacean species endemic to the south-west of Western Australia, which is managed as a wild fishery but also has high aquaculture potential with many commercial facilities under operation. Future studies of *C. cainii* will benefit from the development of polymorphic microsatellite markers from next-generation sequencing (NGS), which can be employed as a tool for the analysis of wild genetic stock structure, captive genetic diversity and genetic improvement (for determining broodstock pedigrees, genetic diversity and inbreeding levels). Polymorphic genetic markers can also be potentially utilised in the conservation of wild *C. tenuimanus* (Smith) stocks, a species commonly known as the hairy marron. This species has become critically endangered mainly due to the introduction of *C. cainii* into the restricted habitat of *C. tenuimanus* [1], and there are concerns that interbreeding and outbreeding depression may cause the extinction of *C. tenuimanus*. To date, 13 microsatellite markers have been developed and used to explore the genetic introgression of *C. cainii* and *C. tenuimanus* [2]. The microsatellite loci developed in the current study provide an important addition to the suite of markers for this genus, especially to improve analyses of parentage and relatedness in captive populations.

Total genomic DNA was extracted using a modified salting out procedure [3] for NGS, from a piece of tissue sourced from the abdomen of one *C. cainii* stored at −80 °C. Two separate samples of DNA template were provided for NGS (2.2 µg and 4.7 µg respectively). In addition, a single walking leg (pereopod) was removed non-destructively from 38 living animals for DNA extraction, for subsequent testing of the developed primers. A 1/8 reaction was prepared for shotgun pyrosequencing on a Roche 454 GS-FLX instrumentation using Titanium chemistry, at the Australian Genome Research Facility (http://www.agrf.com.au). Raw DNA sequence data (46.19 Mbp) was obtained from 184,981 sequence reads, with an average length 250 bp. It was calculated that the reported *C. cainii* data from this study represented approximately 1 % of the average freshwater crayfish genome. Sequences were screened for microsatellites using QDD 2.0 [4], which detected 8799 putative microsatellite loci and PCR primers were designed for 968 microsatellites (695 perfect repeats and 273 compound repeats). Loci were selected based on their suitability for multiplex polymerase chain reaction (PCR) and other key parameters (amplicon length between 100 and 350 bp, minimum length of flanking region 30 bp, maximum number of monobase and di–hexabase repeats in the flanking region of 4 and 2 respectively, and the minimum number of repeats was 5). Optimal primer GC content was set at 50 % (between 40 and 60 %) and at least one consecutive G or C nucleotide was attached to the 3′ end of both the left and right primers (GC clamp). The optimal primer size was set at 20 bp (between 18 and 27 bp) and the optimal primer melting temperature was set at 60 °C (between 57 and 63 °C). Ten microsatellites were selected for primer design and were tested for PCR amplification on 30 captive *C. cainii* broodstock. *C.quadricarinatus* (*n* = 4) and *C. destructor* samples (*n* = 4) were also included for cross-species amplification tests. Prior to PCR, forward primers were tagged with a 5′-M13 universal sequence TGTAAAACGACGGCCAGT [5] and labelled with one of four dyes; FAM, NED, PET or VIC [6]. Genomic DNA (gDNA) was quantified to 50 ng/µL (Nanodrop Technologies ND-1000) for each sample prior to PCR amplification in 10 µL reactions, which included 1 X buffer, 0.2 mM dNTPs, 0.1 mg BSA, 0.5 U mango *Taq* polymerase (Bioline), 1.5 mM MgCl_2_, 0.1 µM of forward primer, 0.2 µM of reverse primer and fluorescently labelled M13 primer. Initially, PCR amplification was carried out separately for each microsatellite locus to determine optimum PCR conditions using a 63–55 °C touchdown program [7]; 94 °C for 3 min, followed by a 32 cycles touchdown (94 °C/20 s; 63 °C down to 55 °C until fifth cycle/45 s; 72 °C/60 s), and 72 °C for 4 min. The PCR products were determined on an ABI 3130 Sequencer (Applied Biosystems) and profiles examined using GENEMAPPER 4.0 (Applied Biosystems) software. Two of the 10 loci failed to amplify for all samples and were subsequently excluded from further analysis. To test for the presence of null alleles, large allele dropout and scoring errors we utilised MICRO-CHECKER 2.2.3 [8], applying 99 % confidence intervals for Monte Carlo simulations. Measures of genetic diversity, such as the number of alleles (*A*), observed (*H*_*o*_) and expected (*H*_*e*_) heterozygosity were estimated with GENALEX 6.5 [9]. The inbreeding coefficient (*F*_*is*_) and tests for Hardy-Weinberg Equilibrium (HWE) were estimated in GENEPOP 4.1 [10] and significance was determined with sequential Bonferroni correction [11]. For the calculation of *F*_*is*_ [12], exact *P*-values under the Markov Chain method were implemented with a dememorization step of 10,000, followed by 20 batches (100 batches for LD) of 5000 iterations per batch. To determine relatedness values and the relationships between captive marron individuals, ML-RELATE was utilised [13]. Dyads were found to comprise a parent offspring, full-sib, half-sib or unrelated relationship.

All *C. cainii* samples amplified at all eight loci and locus *Cca*10 was monomorphic for 30 samples (Additional file 1: Table S1). The average number of alleles per polymorphic locus was 4.7 (ranging from 2 to 14), and overall average *H*_*e*_ was 0.474. Deviations from HWE were detected at two loci (*Cca*06 and *Cca*07). However, given that no null alleles were detected, the latter result is probably attributed to the inbred nature of captive samples [14]. Microsatellite markers have previously been described for *C. cainii* [2], which reported a similar number of alleles (ranging from 2 to 11) and levels of *H*_*e*_ (average *H*_*e*_ = 0.472). From the cross-species amplification tests, *C. destructor* samples amplified at four of the eight loci and *C.quadricarinatus* at two loci. Monomorphic loci were detected at *Cca*09 and *Cca*10 respectively for each species, however, these results should be reassessed with a larger sample size. Upon investigating the relationships between 30 captive *C. cainii* individuals, 79 % of dyads were assigned as unrelated, 9 % parent offspring, 7 % half-sib and 5 % full-sib. Overall relatedness was estimated at 0.12, however, no reference population was available for comparison to relatedness values. Due to the ease of scorability and polymorphic nature of the developed microsatellite loci, the markers are suitable for studies of genetic variation in both wild and captive stocks of *C. cainii*, with potential for cross amplification in other *Cherax* species.

## Availability of supporting data

The microsatellite sequences are available through the National Centre for Biotechnology Information (see http://www.ncbi.nlm.nih.gov/). The following accession numbers are available in the GenBank repository; accession no. KP334240 to KP334247. The full list of microsatellites with designed primers (n = 968) is available at LabArchives DOI 10.6070/H4P26W45.

## Additional file

Additional file 1:
**Table S1.** Characterisation of 8 microsatellite loci for 30 *Cherax cainii* individuals.

## Abbreviations

NGS: next-generation sequencing
PCR: polymerase chain reaction
*A*: number of alleles
*H*_*o*_: observed heterozygosity
*H*_*e*_: expected heterozygosity
*F*_*is*_: inbreeding coefficient
HWE: Hardy–Weinberg equilibrium
