# Supplementary material for: Microsatellite marker development based on next-generation sequencing for the smooth marron (Cherax cainii, Austin) and cross-species amplification in other Cherax species
Source: BMC Res Notes. 2015 Aug 25;8:370. doi: 10.1186/s13104-015-1345-z (PMC4547429; doi:10.1186/s13104-015-1345-z)
Supplement: Additional file 1: — Table S1. Characterisation of 8 microsatellite loci for 30 Cherax cainii individuals. [file 13104_2015_1345_MOESM1_ESM.docx]

Characterisation of 8 microsatellite loci for 30 *Cherax cainii* individuals

Table S1

Number of alleles (*A*), observed (*H_o_*) and expected (*H_e_*) heterozygosity, Hardy-Weinberg Equilibrium (HWE) and inbreeding coefficient (*F_is_*). ^*^significant (*P* < 0.05) following sequential Bonferroni correction. Information represents *C. cainii* unless stated.
